# Determination, measurement, and validation of maximal aerobic speed

**DOI:** 10.1038/s41598-023-31904-1

**Published:** 2023-05-17

**Authors:** Govindasamy Balasekaran, Mun Keong Loh, Peggy Boey, Yew Cheo Ng

**Affiliations:** Human Bioenergetics Laboratory, Physical Education and Sports Science, National Institute of Education, Nanyang Technological University, 1 Nanyang Walk, Singapore, 637616 Singapore

**Keywords:** Cardiovascular biology, Circulation, Metabolism

## Abstract

This study determined Maximal Aerobic Speed (MAS) at a speed that utilizes maximal aerobic and minimal anaerobic contributions. This method of determining MAS was compared between endurance (ET) and sprint (ST) trained athletes. Nineteen and 21 healthy participants were selected for the determination and validation of MAS respectively. All athletes completed five exercise sessions in the laboratory. Participants validating MAS also ran an all-out 5000 m at the track. Oxygen uptake at MAS was at 96.09 ± 2.51% maximal oxygen consumption (${{\dot{\rm{V}}}}\text{O}_{\text{2max}}$). MAS had a significantly higher correlation with velocity at lactate threshold (vLT), critical speed, 5000 m, time-to-exhaustion velocity at delta 50 in addition to 5% velocity at ${{\dot{\rm{V}}}}\text{O}_{\text{2max}}$ (T_lim_υΔ50 + 5%v${{\dot{\rm{V}}}}\text{O}_{\text{2max}}$), and Vsub%95 (υΔ50 or υΔ50 + 5%v${{\dot{\rm{V}}}}\text{O}_{\text{2max}}$) compared with v${{\dot{\rm{V}}}}\text{O}_{\text{2max}}$, and predicted 5000 m speed (R^2^ = 0.90, *p* < 0.001) and vLT (R^2^ = 0.96, *p* < 0.001). ET athletes achieved significantly higher MAS (16.07 ± 1.58 km·h^−1^ vs. 12.77 ± 0.81 km·h^−1^, *p* ≤ 0.001) and maximal aerobic energy (E_MAS_) (52.87 ± 5.35 ml·kg^−1^·min^−1^ vs. 46.42 ± 3.38 ml·kg^−1^·min^−1^, *p* = 0.005) and significantly shorter duration at MAS (ET: 678.59 ± 165.44 s; ST: 840.28 ± 164.97 s, *p* = 0.039). ST athletes had significantly higher maximal speed (35.21 ± 1.90 km·h^−1^, *p* < 0.001) at a significantly longer distance (41.05 ± 3.14 m, *p* = 0.003) in the 50 m sprint run test. Significant differences were also observed in 50 m sprint performance (*p* < 0.001), and peak post-exercise blood lactate (*p* = 0.005). This study demonstrates that MAS is more accurate at a percentage of v${{\dot{\rm{V}}}}\text{O}_{\text{2max}}$ than at v${{\dot{\rm{V}}}}\text{O}_{\text{2max}}$. The accurate calculation of MAS can be used to predict running performances with lower errors (Running Energy Reserve Index Paper).

## Introduction

The measurement of Maximal Aerobic Speed (MAS) is essential for determining aerobic and anaerobic performances of various athletes. However, there is a lack of agreement on the definition and measurement of MAS in existing literature^1^. Terms such as maximal velocity (V_max_), velocity at maximal oxygen uptake (v${{\dot{\rm{V}}}}\text{O}_{\text{2max}}$), peak running velocity, and maximal aerobic velocity have been used to represent MAS. Studies have predominantly considered v${{\dot{\rm{V}}}}\text{O}_{\text{2max}}$ as MAS^1,2^. However, there is a high variability in the literature regarding the speeds and increments used to measure v${{\dot{\rm{V}}}}\text{O}_{\text{2max}}$, which is reported to produce different results for the same runner^3^. Studies on the relative importance of aerobic and anaerobic energy during running have suggested that time to exhaustion (T_lim_) at v${{\dot{\rm{V}}}}\text{O}_{\text{2max}}$ utilizes a higher amount of anaerobic energy and therefore selecting v${{\dot{\rm{V}}}}\text{O}_{\text{2max}}$ as MAS may not be accurate^4–6^. Since MAS should utilize maximal aerobic energy (E_MAS_) and minimal possible anaerobic energy contribution, MAS should be lower than v${{\dot{\rm{V}}}}\text{O}_{\text{2max}}$ at a precise speed with a corresponding lower blood lactate (BLa) response^1^. In addition, there is a wide range of intergroup variation in maximal oxygen uptake (${{\dot{\rm{V}}}}\text{O}_{\text{2max}}$) between individuals, which vary according to the athletic background and gender of the athlete^7^. Hence, there is currently no universal acceptance of a single standard of measure of MAS.

Exercising above critical speed (CS), which is close to the velocity of lactate threshold (vLT), leads to slow additional increases of oxygen uptake (${{\dot{\rm{V}}}}\text{O}_{\text{2}}$)^8^. Lactate threshold (LT) is usually detected at the point where BLa has a nonlinear increase during exercise as it reflects net lactate production that had exceeded lactate elimination. Such BLa concentrations are usually taken during graded incremental exercise tests that indicate lactate curves. Therefore, the shift in lactate curves indicate a change in aerobic capacity, also known as LT^9^. This slow component of ${{\dot{\rm{V}}}}\text{O}_{\text{2}}$ becomes apparent at approximately 80–110 s from the start of maximal effort exercise, where a range of speeds is estimated as E_MAS_^10^. One of the proposed intensities at which E_MAS_ can be determined is known as velocity of delta 50 (υΔ50), the median of v${{\dot{\rm{V}}}}\text{O}_{\text{2max}}$ and vLT^11^. Measurements for vLT, v${{\dot{\rm{V}}}}\text{O}_{\text{2max}}$, and υΔ50 of 8 highly trained long distance runners found υΔ50 to be at 91% of ${{\dot{\rm{V}}}}\text{O}_{\text{2max}}$ (${{\dot{\rm{V}}}}\text{O}_{\text{2max}}$ = 59.8 ml·kg^−1^·min^−1^, v${{\dot{\rm{V}}}}\text{O}_{\text{2max}}$ = 18.5 km·h^−1^, vLT = 15.2 km·h^−1^, υΔ50 = 16.9 km·h^−1^)^12^. However, this speed did not seem to elicit E_MAS_ in trained athletes^8^. Hence, a hypothetical minimum intensity of υΔ50 + 5%v${{\dot{\rm{V}}}}\text{O}_{\text{2max}}$ will be used in this study for participants who did not achieve E_MAS_ at υΔ50.

Anaerobic energy utilization is estimated as the time spent at ${{\dot{\rm{V}}}}\text{O}_{\text{2max}}$ during T_lim_v${{\dot{\rm{V}}}}\text{O}_{\text{2max}}$. This is based on the assumption that anaerobic energy stores will be completely depleted during T_lim_ at intensities above CS^13^. This has been demonstrated in previous studies assuming that maximal anaerobic energy (E_MAnS_) was consumed during 800–5000 m^14^ as well as 1500–10,000 m^15^ runs. It is necessary to select the intensity at which the consumed anaerobic energy is a representative of the anaerobic energy used at any run with an aerobic speed reserve (AeSR), where AeSR represents the difference between v${{\dot{\rm{V}}}}\text{O}_{\text{2max}}$ and CS^16^. MAS lies at the extreme of the range between CS and v${{\dot{\rm{V}}}}\text{O}_{\text{2max}}$. During T_lim_v${{\dot{\rm{V}}}}\text{O}_{\text{2max}}$, the athlete attains E_MAS_ and uses E_MAnS_ with minimal aerobic contribution. T_lim_${{\dot{\rm{V}}}}\text{O}_{\text{2max}}$ determined at other intensities within this range may consume comparatively higher percentage of ${{\dot{\rm{V}}}}\text{O}_{\text{2}}$ and thus overestimate the anaerobic energy. Hence, T_lim_${{\dot{\rm{V}}}}\text{O}_{\text{2max}}$v${{\dot{\rm{V}}}}\text{O}_{\text{2max}}$ as anaerobic energy seems logical to measure duration at MAS (MAS_dur_) and MAS.

To determine MAS and MAS_dur_, anaerobic energy consumption at MAS has to be minimized without compromising its criteria. MAS_dur_ can be calculated by subtracting anaerobic energy duration from ${{\dot{\rm{V}}}}\text{O}_{\text{2max}}$ till exhaustion at Vsub%95 (T_lim_Vsub%95). This method was based on the negative linear relationship between anaerobic and aerobic energy contribution during physical activity, as anaerobic energy contribution decreases with increasing exercise duration^17^. Therefore, subtracting anaerobic energy duration from T_lim_Vsub%95 may provide an accurate determination of MAS_dur_.

The objectives of this study aimed to (1) determine MAS at a speed that utilizes maximal aerobic and minimal anaerobic contributions, where MAS should fulfill four criteria (a) MAS should be lower than v${{\dot{\rm{V}}}}\text{O}_{\text{2max}}$, (b) maximal aerobic energy utilization is elicited during T_lim_ test, (c) MAS should occur at a specific percentage fraction of v${{\dot{\rm{V}}}}\text{O}_{\text{2max}}$, and (d) estimated anaerobic energy contribution at T_lim_MAS should be lower than that at T_lim_v${{\dot{\rm{V}}}}\text{O}_{\text{2max}}$. (2) To assess whether MAS can accurately differentiate between athletes of different training orientations (endurance or sprint trained) and if there was an association between MAS_dur_ and aerobic performance variables of run distance and best performance times. It was hypothesized that the MAS of endurance-trained athletes would be higher than that of sprint-trained athletes, and that MAS measured would significantly correlate with 5000 m run performance and aerobic performances variables. This study has been separated into two parts. The first part of this study, which this paper is based on, utilizes a new framework of calculating MAS. This validated MAS was confirmed with the prediction of running performances in a follow-up paper that examined the Running Energy Reserve Index (RERI)^18^.

## Methods

### Participants

Forty participants volunteered for the study. Among the 40 athletes, 19 healthy participants (age: 29.74 ± 8.31 years; height: 171.86 ± 7.65 cm; body mass index (BMI): 22.01 ± 2.12 kg·m^−2^; body fat percentage (BF%): 12.96 ± 3.10%)) were selected to validate the theoretical framework criteria of MAS. The remaining participants consisted of 9 sprint-trained athletes (age: 26.89 ± 9.39 years; height: 174.16 ± 5.69 cm; BMI: 23.09 ± 2.07 kg·m^−2^; BF%: 10.59 ± 2.55%) and 12 endurance-trained athletes (age: 31.67 ± 7.24 years; height: 173.67 ± 7.59 cm; BMI: 21.34 ± 1.27 kg·m^−2^; BF%: 12.74 ± 2.38%) (Table 1). These 21 athletes were selected to determine whether there were significant differences between the MAS of sprint-trained and endurance-trained athletes, and the relation of MAS with aerobic performances and variables.

**Table 1:** Descriptive characteristics endurance-trained and sprint-trained athletes.

Variables	Endurance-trained	Sprint-trained
N	12	9
Age (years)	31.67 ± 7.24	26.89 ± 9.39
Height (cm)	173.67 ± 7.59	174.16 ± 5.69
BMI (kg·m^−2^)	21.34 ± 1.27	23.09 ± 2.07*
Fat percentage (%)	12.74 ± 2.38	10.59 ± 2.55
Hematocrit (%)	43.51 ± 2.30	45.59 ± 1.34*
Haemoglobin (g·dl^−1^)	14.79 ± 0.78	15.50 ± 0.46*
Plasma volume (%)	56.49 ± 2.30	54.41 ± 1.34*
LBM (kg)†	53.13 ± 5.50	58.76 ± 4.43*
FFM (kg)†	55.68 ± 5.80	61.69 ± 4.65*
BMD (g·cm^−2^)†	1.19 ± 0.07	1.28 ± 0.10*
BMC (kg)†	2.61 ± 0.23	2.93 ± 0.29*
Rest BLa (mmol·L^−1^)	0.71 ± 0.13	0.83 ± 0.16

Values are in means ± SD. *BMI* Body mass index, *LBM* Lean body mass, *FFM* Fat free mass, *BMD* Bone mineral density, *BMC* Bone mineral content, *BLa* Blood lactate.

*p ≤ 0.05, **p ≤ 0.01: Indicates significant difference between endurance-trained and sprint-trained athletes.

^†^The data of two participants aged 14.5 ± 0.5 years were not included due to differences between age of these two participants and the total cohort and its effect on body composition^19^ (Boileau and Horswill 2003^20^).

Participants were considered trained if they were engaged in training for at least four sessions of 60 min per week in their chosen activities for the last 12 months. Among the endurance-trained athletes, 4 were triathletes and had completed the ironman distance race (3.86 km swim, 180.25 km bike, and 42.195 km run) several times. The other 6 participants were training for half and full marathon, and the remaining 2 were 10 km runners. The sprint-trained athletes were specialized in soccer and 100–400 m sprint events, and they were still actively competing in their respective events. Participants who had any history of musculoskeletal injuries in the past 6 months, smokers and medical history were exempted from this study. All participants were informed of the risk and benefits of the study and gave their informed consent to participate. This study was approved by the Ethical Review Board of the Research and Graduate Studies Committee of Physical Education & Sports Science, National Institute of Education, Nanyang Technological University, Singapore. All methods were performed in accordance with the relevant guidelines, regulations and STROBE checklist.

### Experimental design

The experimental design and procedures in this study were derived and modified from Bundle et al.^21^. A within cross-sectional design was utilized in each investigation, where each participant underwent a series of exercise tests to determine MAS accurately. Participants completed exercise sessions which included (1) aerobic metabolic measurement utilizing Astrand modified running (AMRMAX) continuous incremental maximal treadmill protocol, (2) submaximal discontinuous treadmill run (SUBMAX) protocol, (3) T_lim_ at v${{\dot{\rm{V}}}}\text{O}_{\text{2max}}$, (4) Test of T_lim_ at Vsub%95, and (5) speed and duration test protocols. To assess the validity of MAS, participants also ran an all-out 5000 m on the track. Participants were instructed to avoid strenuous activities, alcohol, and caffeine 24 h before testing.

All laboratory sessions were conducted at the Human Bioenergetics Laboratory in the Physical Education and Sports Science department of the National Institute of Education, Nanyang Technological University, Singapore, while the 5000 m track test was performed on the 400 m track located at the Sports and Recreation Centre of Nanyang Technological University, Singapore.

### Pretest preparations

Prior to the tests where cardiorespiratory and aerobic metabolic parameters were measured, the flow meter, sampling line and gas calibrations of ParvoMedics TrueOne 2400 (ParvoMedics Inc, UT, USA) were performed according to the procedures explained in the instruction manual (Operator’s guide, Version 4.3, ParvoMedics Inc, UT, USA 2008). Heart rate (HR) transmitters were strapped onto the participants’ chest, and participants were required to put on the head cap, mouthpiece of a two-way non-rebreathing valve. A nose-clip was used to ensure all expired air are analyzed. In addition, participants were strapped in an upper body safety harness to prevent falling while running on the treadmill belt at various speeds. The harness did not assist or impede the participants during the tests.

### Experimental tests and measurements

Participants were instructed to stride the belt of the treadmill before the tests, and to hold the handrail of the treadmill or give a ‘thumbs down’ signal to stop the test due to exhaustion or discomfort. All the laboratory tests were performed on a motorized treadmill (H-P Cosmos, UK). The gradient was set at 1% for all treadmill running protocols except for ${{\dot{\rm{V}}}}\text{O}_{\text{2max}}$ protocol^22^. Participants were encouraged to deliver their maximum effort during tests.

Before performing the ${{\dot{\rm{V}}}}\text{O}_{\text{2max}}$ test, height and weight of participants were recorded, and a Dual-Energy X-ray absorptiometry (DEXA, QDR 4500W, Hologic Inc, Waltham, USA) scan was performed to determine body composition. Subsequently, capillary blood sample was collected via the finger prick technique to measure resting BLa.

#### Astrand modified running continuous incremental maximal treadmill (AMRMAX) protocol

The AMRMAX protocol was employed to determine ${{\dot{\rm{V}}}}\text{O}_{\text{2max}}$ of participants. The test began with an initial speed of 8–12 km·h^−1^ with 0% gradient. After 3 min of running, the gradient was increased by 2.5% at 2 min stages until volitional exhaustion. Thereafter, post-exercise capillary whole blood samples were taken from the finger at every minute for 5 min. BLa was analyzed via YSI 2300 STAT Plus (2300 D, YSI Incorporated, USA) to measure peak post-exercise BLa. The expired breath-by-breath gas concentrations were analyzed using ParvoMedics TrueOne 2400 (ParvoMedics, Inc, USA) and averaged at every 15 s. HR was measured via a Polar HR transmitter (Polar Electro, Singapore) which sends its signals to the receiver of ParvoMedics TrueOne 2400 metabolic system (ParvoMedics, Inc, USA).

${{\dot{\rm{V}}}}\text{O}_{\text{2max}}$ was determined when participants satisfied three of the following five criteria^23^: (1) Plateau of ${{\dot{\rm{V}}}}\text{O}_{\text{2}}$ change in ${{\dot{\rm{V}}}}\text{O}_{\text{2}}$ ≤ 2.1 ml·kg^−1^·min^−1^ in spite of increasing treadmill gradient, (2) Respiratory exchange ratio (RER) at ${{\dot{\rm{V}}}}\text{O}_{\text{2max}}$ ≥ 1.1, (3) BLa > 8 mmol·L^−1^, (4) HR ≥ 90% of the age predicted maximal HR (HR_max_), and (5) volitional exhaustion^9^.

#### Submaximal discontinuous treadmill (SUBMAX) protocol

Participants performed a series of six to nine discontinuous submaximal treadmill runs. Initial speed was set at approximately 40–60% ${{\dot{\rm{V}}}}\text{O}_{\text{2max}}$ with increments of 4–5% ${{\dot{\rm{V}}}}\text{O}_{\text{2max}}$ at every stage depending on the ability of the participant. All running speeds were within the range of 40–90% ${{\dot{\rm{V}}}}\text{O}_{\text{2max}}$. Running sessions were fixed at 4 min^23,24^, with 2–4 min recovery between sessions. Capillary blood samples were obtained with the finger prick technique and were collected immediately after each submaximal running session. Steady state cardiorespiratory and aerobic metabolic measures were recorded at every 15 s during the 3rd and 4th minute of each treadmill running session.

vLT was then determined using a log–log plot method^25,26^. The linear relation between run speeds and corresponding ${{\dot{\rm{V}}}}\text{O}_{\text{2}}$ were determined using a linear regression analysis^21,27,26^. Linear relation determined through SUBMAX protocol was extrapolated to ${{\dot{\rm{V}}}}\text{O}_{\text{2max}}$, and this velocity at ${{\dot{\rm{V}}}}\text{O}_{\text{2max}}$ was termed as v${{\dot{\rm{V}}}}\text{O}_{\text{2max}}$^26^. The average of vLT and v${{\dot{\rm{V}}}}\text{O}_{\text{2max}}$ was calculated to determine υΔ50.

#### Oxygen Consumption till Exhaustion (${{\dot{\rm{V}}}}\text{O}_{\text{2max}}$ till exhaustion (T_lim_)) tests

T_lim_ tests were conducted at 100% v${{\dot{\rm{V}}}}\text{O}_{\text{2max}}$ (T_lim_v${{\dot{\rm{V}}}}\text{O}_{\text{2max}}$) and υΔ50. However, it was found that the participants could not reach ${{\dot{\rm{V}}}}\text{O}_{\text{2max}}$ at υΔ50. Hence, 5%v${{\dot{\rm{V}}}}\text{O}_{\text{2max}}$ was added to υΔ50 for all participants to achieve maximal aerobic energy during the T_lim_ test (υΔ50 ± 5%v${{\dot{\rm{V}}}}\text{O}_{\text{2max}}$). The speed at which E_MAS_ was attained during T_lim_ at υΔ50 and υΔ50 ± 5%v${{\dot{\rm{V}}}}\text{O}_{\text{2max}}$ was termed Vsub%95. Achieving ≥ 95% ${{\dot{\rm{V}}}}\text{O}_{\text{2max}}$ was selected as the primary criterion to measure time to attain ${{\dot{\rm{V}}}}\text{O}_{\text{2max}}$ (TA${{\dot{\rm{V}}}}\text{O}_{\text{2max}}$) during T_lim_v${{\dot{\rm{V}}}}\text{O}_{\text{2max}}$ and T_lim_Vsub%95^27,19^.

Participants performed a warm up protocol of 8–15 min at 60% ${{\dot{\rm{V}}}}\text{O}_{\text{2max}}$ followed by a rest interval of 5–10 min. During each of the T_lim_ test, participants ran at a fixed speed for as long as possible until volitional exhaustion. Breath-by-breath cardiorespiratory and aerobic metabolic measures were recorded during each run. BLa samples were collected after warm up and at each minute of the first five minutes after individual T_lim_ run to determine peak post-exercise BLa.

Breath-by-breath ${{\dot{\rm{V}}}}\text{O}_{\text{2}}$ responses recorded at T_lim_v${{\dot{\rm{V}}}}\text{O}_{\text{2max}}$ were interpolated per second and the time was aligned to the start of the run with an average at every five seconds via a moving average filter. Thereafter, the data was fitted to a positive exponential nonlinear regression by means of weighted least square method using SigmaPlot software (windows version 11.0.0.77, Germany) (Eq. 1). This equation was fitted to the data collected from T_lim_ tests and TA${{\dot{\rm{V}}}}\text{O}_{\text{2max}}$ and T_lim_${{\dot{\rm{V}}}}\text{O}_{\text{2maxconverted}}$ were computed (Eqs. 2 and 3).

1 $$\dot{V}{\text{O}}_{2} \left( {\text{t}} \right) = {\dot{\text{V}}\text{O}}_{{2{\text{ baseline}}}} + {\text{A}}_{0} \times \left[ {1 - {\text{e}}^{{ - \left( {\frac{{\text{t}}}{{{\uptau }_{0} }}} \right)}} } \right] + {\text{A}}_{1} \times \left[ {1 - {\text{e}}^{{ - \left( {\frac{{{\text{t}} - {\updelta }_{1} }}{{{\uptau }_{1} }}} \right)}} } \right]$$

where ${{\dot{\rm{V}}}}\text{O}$_2baseline_ is the ${{\dot{\rm{V}}}}\text{O}$_2_ before starting the T_lim_ run, A is the amplitude of ${{\dot{\rm{V}}}}\text{O}$_2_ (${{\dot{\rm{V}}}}\text{O}$_2max_–${{\dot{\rm{V}}}}\text{O}$_2baseline_) for I, and II components, δ is the time delay before onset of each exponential component and τ is the time constant for each component of ${{\dot{\rm{V}}}}\text{O}$_2_^28^.

2 $$T_{\lim } {\dot{V}}O_{2\max } v\dot{V}O_{2\max } = T_{\lim } v\dot{V}O_{2\max } {-} TA\dot{V}O_{2\max } v\dot{V}O_{2\max }$$

3 $${\text{T}}_{\lim } {\dot{\text{V}}\text{O}}_{{2\max {\text{converted}}}} \left( s \right) = \frac{{\left( {T_{\lim } {\dot{\text{V}}\text{O}}_{2\max } v{\dot{\text{V}}\text{O}}_{2\max } \times v{\dot{\text{V}}\text{O}}_{2\max } } \right)}}{{V{\text{sub}}\% 95 }}$$

#### Speed and duration curve protocol

After pretest preparations, orientation trials were conducted by allowing participants to step onto the treadmill at fast speeds. Following a 5–10min recovery, the treadmill was set at a preselected speed. Participants then stepped on the moving treadmill with the use of the handrail and started unassisted running within 4–7 steps. They were instructed to run until volitional exhaustion, and both duration and run speeds at exhaustion were recorded. Full recovery was given between the trials, and they were allowed to discontinue the test if they were unable to perform at their best. A minimum of two to three trials were performed at different speeds ranged from 110% v${{\dot{\rm{V}}}}\text{O}$_2max_ to 140% v${{\dot{\rm{V}}}}\text{O}$_2max_. Participants were only allowed to perform the next trial if: (1) recovery HR was equal to or more than 120 beats·min^−1^ approximately, (2) participant gave consent for performing the test to the best of their abilities, and (3) duration of recovery was based on the principle of work to rest ratio.

**Figure 1:**
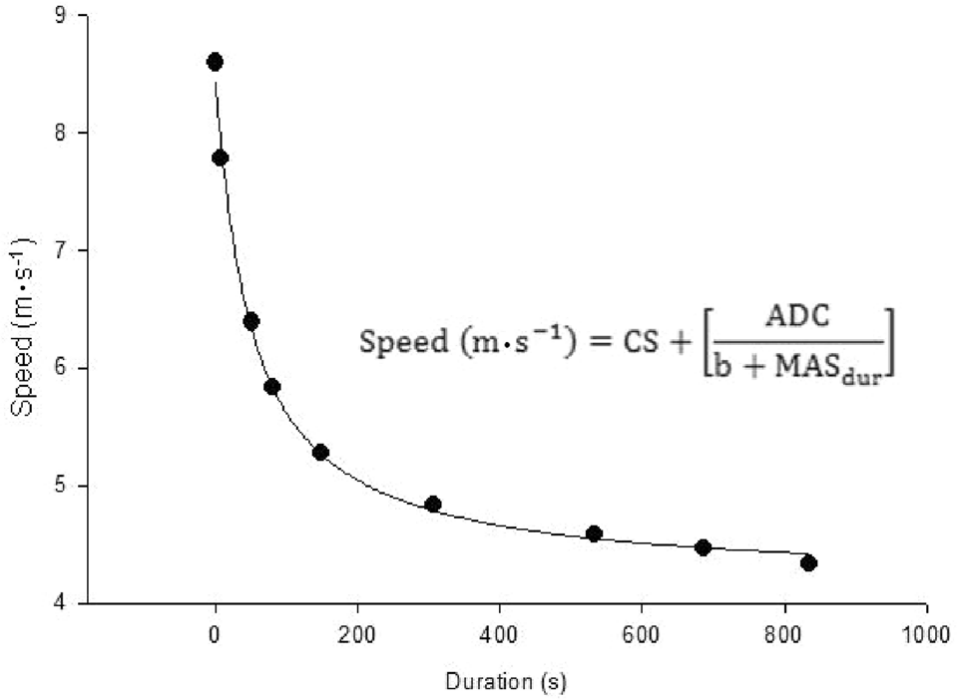
Hyperbolic relationship between speed and duration.

Speeds in the range of 90–140% v${{\dot{\rm{V}}}}\text{O}$_2max_ and their corresponding durations calculated during the different T_lim_ sessions and speed-duration curve protocol were data fitted to determine hyperbolic relation (Fig. 1). MAS was then determined using Eq. 4.

4 $${\text{Speed }}\left( {{\text{m}\cdot {s}}^{ - 1} } \right) = {\text{CS}} + \left[ {\frac{{{\text{ADC}}}}{{{\text{B}} + {\text{MAS}}_{{{\text{dur}}}} }}} \right]$$

where CS = critical speed; ADC = anaerobic distance capacity; MAS_dur_ = duration at MAS; and B = constant.

A backward validation by predicting run performances was performed whereby MAS_dur_ was calculated by adding the time representing anaerobic energy^18^. Since there is a negative relationship between aerobic and anaerobic energy, aerobic energy was taken to be the negative of anaerobic energy. The following equation was employed for the calculation of MAS_dur_ (Eq. 5):

5 $${\text{MAS}}_{dur} = T_{\lim } Vsub\% 95 {-} \left( {{-} T_{\lim } \dot{V}O_{2\max converted} } \right)$$

The linear relation between speed and ${{\dot{\rm{V}}}}\text{O}$_2_ (measured through the SUBMAX protocol) was extrapolated to MAS and the extrapolated ${{\dot{\rm{V}}}}\text{O}$_2_ at MAS was considered as E_MAS_^21^.

#### 50 m sprint run test

Participants performed a general 10–15 min warm up run at a comfortable pace followed by dynamic stretching exercises. Following the warm up, participants performed strides of 20–40 m with 3–5 min recovery between strides.

The 50 m sprint run was performed with a standing start position at the start line. At the start command, the athlete accelerated and covered the distance of 50 m in the least possible time. The speed and time at the stipulated distance intervals within 50 m were automatically recorded by the five timing gates placed within 34–50 m for sprinters and middle distance runners and within 30–46 m for endurance athletes. A minimum of two trials were performed, with a 15–20 min rest interval in between the trials, and the best performance was recorded to the nearest 0.01 s.

#### 5000 m test

Orientation trials were performed 1 week before testing to familiarize participants with the pace of their run to elicit the best effort in testing. Prior to the actual run, participants warmed up for 10–15 min at a comfortable pace followed by stretching exercises. A rest period of 3–5 min after the warm up was given before starting the test. Participants were encouraged to run at their targeted best effort based on their fitness level and ran the whole distance at their own self-regulated pace. The time taken to cover each run was recorded to the nearest 0.01 s.

### Statistical analysis

Statistical analyses and data fitting procedures were performed using Statistical Package for Social Sciences (SPSS) version 17.0 and SigmaPlot software (version 11.0, Systat software, Inc., 2008, Germany) respectively. Using a power of 0.80 and α level of 0.05 with an effect size of > 1.1, it was determined that a minimum of 10 participants were required^29^. Linear regression was employed to calculate vLT, v${{\dot{\rm{V}}}}\text{O}$_2max_, and E_MAS_. One-way ANOVA was utilized to measure any significant differences between BLa measured during the different T_lim_ tests and BLa measured at ${{\dot{\rm{V}}}}\text{O}$_2max_ $({\text{BLa}}_{{{{\dot{{\rm V}}{\rm O}}}2\max }} )$. The Wilcoxon rank test (non-parametric paired t-test) and correlation technique were employed to significantly validate the criteria of MAS, and independent t-tests were employed to compare anthropometrical and body composition measures, cardiorespiratory and aerobic metabolic measures, and MAS between endurance-trained and sprint-trained athletes. Lastly, coefficient of correlation technique (very strong correlation: 0.9–1.0, strong correlation: 0.7–0.9, moderate correlation: 0.5–0.7) was used to assess the relationship between MAS and aerobic parameters. Statistical significance was set at *p* ≤ 0.05 for this study.

## Results

**Figure 2:**
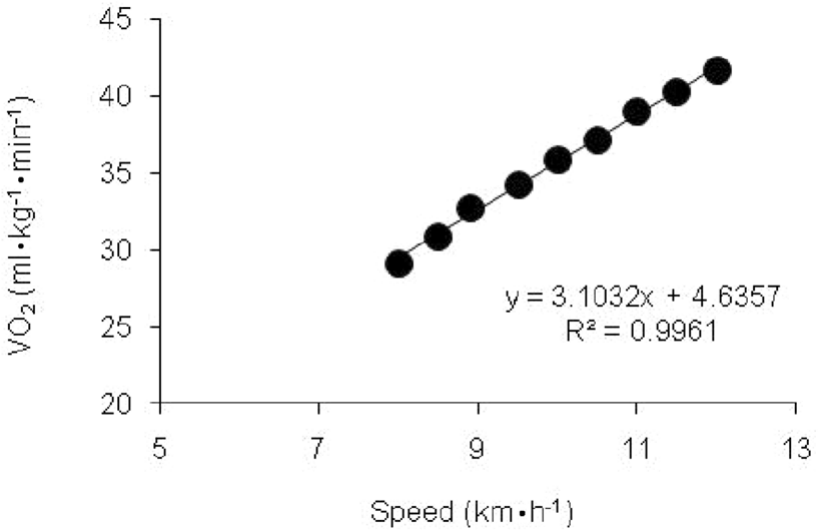
Determination of the submaximal efficiency equation between ${{\dot{\rm{V}}}}\text{O}$_2_ and corresponding run speeds.

As shown in Table 2, anthropometrical, body composition, and hematological measures were significantly higher among sprint-trained athletes compared to endurance-trained athletes. However, the proportion of plasma volume was significantly higher among endurance-trained athletes. Figure 2 determined the steady state of the participants during the SUBMAX protocol calculated by the submaximal efficiency equation.

**Table 2:** Astrand Modified Running Protocol (AMRMAX) results in endurance-trained and sprint-trained athletes.

Variables	Endurance-trained	Sprint-trained
N	12	9
${{\dot{\rm{V}}}}\text{O}_{\text{2max}}$ (ml·kg ^−1^·min^−1^)	57.62 ± 5.40	51.12 ± 3.59**
RER_max_	1.16 ± 0.03	1.12 ± 0.03^a^ **
${\text{HR}}_{{{\dot{\rm{V}}}}\text{O}_{\text{2max}}}$ (beats·min^-1^)	181.71 ± 14.31	185.06 ± 5.81
%HR_max_ at ${{\dot{\rm{V}}}}\text{O}_{\text{2max}}$	96.45 ± 6.02	95.89 ± 5.96
${\text{BLa}}_{{{\dot{\rm{V}}}}\text{O}_{\text{2max}}}$ (mmol·L^−1^)	8.26 ± 1.72	8.17 ± 1.63

Values are in means ± SD.

${{\dot{{V}}}}{O}$_*2max*_ Maximal oxygen uptake, *RER*_*max*_ Respiratory exchange ratio at ${{\dot{\rm{V}}}}\text{O}$_2max_, $HR_{{\dot{{V}}}}$_*O2max*_ Heart rate at ${{\dot{\rm{V}}}}\text{O}$_2max_, %*HR*_*max*_ Percentage of maximal heart rate, $BLa_{{\dot{{V}}}}$_*O2max*_ Blood lactate at ${{\dot{\rm{V}}}}\text{O}$_2max_.

*p < 0.05, **p < 0.01: Indicates significant difference between endurance-trained and sprint-trained athletes.

^a^Only 8 sprint trained participants were analyzed due to technical difficulties.

### MAS_dur_ calculation

**Figure 3:**
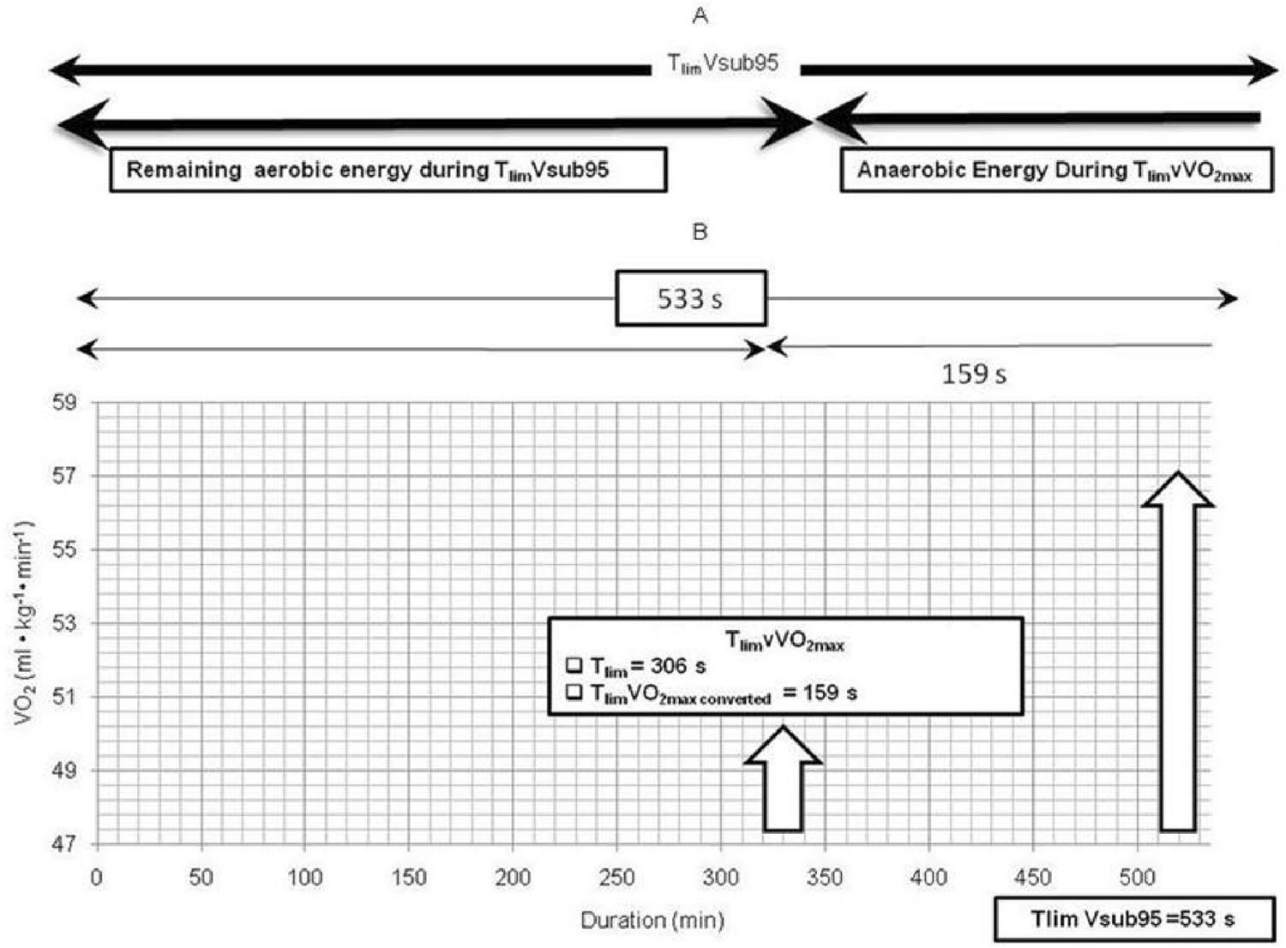
Calculation of duration at MAS. (**A**) indicates the duration of MAS with anaerobic and aerobic energy. (**B**) indicates the calculation of MAS based on duration of T_lim_Vsub%95 and T_lim_${{\dot{\rm{V}}}}\text{O}_{\text{2maxconverted}}$ at T_lim_v${{\dot{\rm{V}}}}\text{O}$_2max_.

MAS_dur_ was calculated by subtracting T_lim_${{\dot{\rm{V}}}}\text{O}_{\text{2maxconverted}}$ from T_lim_Vsub%95. This however resulted in MAS being higher than Vsub%95, which elicited higher anaerobic energy and thus failed to fulfil the MAS criteria. Figure 3 shows an example of a participant whose T_lim_${{\dot{\rm{V}}}}\text{O}_{\text{2maxconverted}}$ and T_lim_Vsub%95 were at 159 s and 533 s respectively. Subtracting these two would have resulted in a corresponding speed at MAS_dur_ of 16.9 km·h^−1^ on the speed-duration graph ((306 s = 5 min 6 s (16.9 km·h^-1^) → converted to T_limconverted_ = 159 s (using Eq. 3). T_lim_Vsub95 = 533 s—(− T_limconverted_) 159 s = 692 s (MAS_dur_) (using Eq. 5), 692 s = 11 min 32 s)). This translated to 97.1%v${{\dot{\rm{V}}}}\text{O}$_2max_, which was close to v${{\dot{\rm{V}}}}\text{O}$_2max_ at which E_MAnS_ was determined.

Using the same participant in Fig. 3, adding T_lim_${{\dot{\rm{V}}}}\text{O}_{\text{2maxconverted}}$ and T_lim_Vsub%95 together resulted in a corresponding MAS_dur_ speed at 16.1 km·h^−1^, which was at 92.5%v${{\dot{\rm{V}}}}\text{O}_{\text{2max}}$. It seemed that T_lim_${{\dot{\rm{V}}}}\text{O}_{\text{2maxconverted}}$ and T_lim_Vsub%95 fulfilled the criteria of achieving MAS. This suggest that accurate calculation of MAS will result in lower error of prediction of run performances with an average of 2.39 ± 2.04% (R^2^ = 0.99, n_T_ (number of running trials) = 252)) for all athletes, with treadmill trials to within an average of 2.26 ± 1.89% (R^2^ = 0.99, n_T_ = 203) and track trials to within an average of 2.95 ± 2.51% (R^2^ = 0.99, n_T_ = 49)^18^.

### Validation of MAS

The mean MAS was 14.50 ± 1.82 km·h^−1^. There was no significant difference between ${{\dot{\rm{V}}}}\text{O}$_2_ at MAS (96.09 ± 2.51% ${{\dot{\rm{V}}}}\text{O}$_2max_) and ${{\dot{\rm{V}}}}\text{O}$_2_ at 95% ${{\dot{\rm{V}}}}\text{O}$_2max_ among all athletes (${{\dot{\rm{V}}}}\text{O}$_2_ at MAS: 50.18 ± 5.19 ml·kg^−1^·min^−1^ vs. ${{\dot{\rm{V}}}}\text{O}$˙_2_ at 95% ${{\dot{\rm{V}}}}\text{O}$_2max_: 50.69 ± 4.69 ml·kg^−1^·min^−1^, *p* = 0.134). In addition, mean BLa at MAS (BLa_MAS_) (7.80 ± 1.52 mmol·L^−1^) was significantly lower than corresponding values at v${{\dot{\rm{V}}}}\text{O}$_2max_ (9.11 ± 2.50 mmol·L^−1^; *p* = 0.009) and ${{\dot{\rm{V}}}}\text{O}$_2max_ (8.60 ± 1.62 mmol·L^−1^; *p* = 0.037**)**. While BLa_MAS_ was not significantly lower than BLa at Vsub%95 (BLa_Vsub%95_) (8.01 ± 1.39 mmol·L^−1^, *p* = 0.174). RER, ventilatory threshold and HR at MAS were 1.05 ± 0.03, 2.19 ± 0.51 L·min^−1^ and 176.62 ± 26.72 beats·min^−1^ respectively.

### MAS between endurance-trained and sprint-trained athletes

Endurance-trained athletes had significantly higher mean ${{\dot{\rm{V}}}}\text{O}$_2max_ (*p* = 0.004) and RER at ${{\dot{\rm{V}}}}\text{O}$_2max_ (RER_max_) (*p* = 0.007) (Table 2). vLT (*p* < 0.001), BLa at LT (BLa_LT_) (*p* < 0.001), ${{\dot{\rm{V}}}}\text{O}$_2_ at LT (${{\dot{\rm{V}}}}\text{O}$_2LT_) (*p* = 0.013) were significantly higher among ET athletes, while no significant differences were observed between both cohorts for HR at LT (HR_LT_) (*p* = 0.467) and percentage of HR_max_ (%HR_max_) (*p* = 0.968) (Table 3). In addition, measured υΔ50 (*p* < 0.001) and υΔ50 + 5%v${{\dot{\rm{V}}}}\text{O}$_2max_ (*p* < 0.001) were also significantly higher in endurance-trained athletes compared to sprint-trained athletes (Table 4).

**Table 3:** Submaximal discontinuous treadmill run (SUBMAX) test results in endurance-trained and sprint-trained athletes.

Variables	Endurance-trained	Sprint-trained
N	12	9
vLT (km·h^−1^)	13.37 ± 1.58	10.48 ± 0.83***
BLa_LT_ (mmol·L^−1^)	1.59 ± 0.59	2.60 ± 0.49***
${{\dot{\rm{V}}}}\text{O}$_2LT_ (ml·kg^−1^·min^−1^)	43.63 ± 4.15	38.76 ± 3.92*
HR_LT_ (beats·min^-1^)	157.83 ± 15.42	161.96 ± 7.14
HR_LT_ (%HR_max_)	83.73 ± 6.60	83.83 ± 4.20
υΔ50 (km·h^−1^)	15.37 ± 1.57	12.42 ± 0.81***
υΔ50 + 5%v${{\dot{\rm{V}}}}\text{O}$_2max_ (km·h^−1^)	16.25 ± 1.64	13.12 ± 0.85***

Values are in means ± SD.

*vLT* velocity at lactate threshold, *BLa*_*LT*_ Blood lactate at LT, ${{\dot{{V}}}}{O}$_*2LT*_ Oxygen uptake at LT, *HR*_*LT*_ Heart rate at LT, %*HR*_*max*_  Percentage of maximal heart rate; υΔ50 median of v${{\dot{{V}}}}{O}$_2max_ and vLT, *υΔ50 or υΔ50 + 5%v*${{\dot{{V}}}}{O}$_*2max*_ mean speed of v${{\dot{\rm{V}}}}\text{O}$_2max_ and vLT or mean speed of v${{\dot{\rm{V}}}}\text{O}$_2max_ and vLT plus 5%v${{\dot{\rm{V}}}}\text{O}$_2max_.

*p < 0.05, **p < 0.01, ***p < 0.001: Indicates significant difference between endurance-trained and sprint-trained athletes.

**Table 4:** Oxygen kinetics and blood lactate at v${{\dot{\rm{V}}}}\text{O}$_2max_ and Vsub%95 among endurance-trained and sprint-trained athletes.

Variables	Endurance- trained_v_${{\dot{\rm{V}}}}\text{O}$_2max_	Sprint- trained_v_${{\dot{\rm{V}}}}\text{O}$_2max_	Endurance-trained_Vsub%95_	Sprint-trained_Vsub%95_
Speed (km·h^−1^)	17.38 ± 1.62	14.33 ± 1.11***	16.25 ± 1.64	13.05 ± 0.82***
T_lim_ (s)	300.02 ± 67.43	358.50 ± 36.70*	552.84 ± 105.50	672.29 ± 120.98*
TA${{\dot{\rm{V}}}}\text{O}$_2max_ (s)	182.61 ± 34.57	206.07 ± 39.85	356.89 ± 67.59^a^	367.31 ± 120.52
T_lim_${{\dot{\rm{V}}}}\text{O}_{\text{2max}}$ (s)	117.41 ± 71.47	152.43 ± 45.17	215.77 ± 127.80^a^	304.98 ± 114.18
T_lim_${{\dot{\rm{V}}}}\text{O}_{\text{2maxconverted}}$ (s)	125.75 ± 76.28	167.98 ± 52.28*	–	–
BLa (mmol·L^-1^)	8.96 ± 1.87	7.96 ± 1.68	7.97 ± 1.59	7.27 ± 1.25

Values are presented as means ± SD.

*v*${{\dot{{V}}}}{O}$_*2max*_ Velocity at ${{\dot{\rm{V}}}}\text{O}$_2max_, *Vsub%95* speed at υΔ50 or υΔ50 + 5%v${{\dot{\rm{V}}}}\text{O}$_2max_ at which maximal aerobic energy was obtained, *T*_*lim*_ ${{\dot{\rm{V}}}}\text{O}$_2_ till exhaustion, *TA*${{\dot{{V}}}}{O}$_*2max*_ Time to achieve 95%${{\dot{{V}}}}{O}$_2max_, *T*_*lim*_${{\dot{\rm{V}}}}\text{O}$_*2max*_ time spent at ${{\dot{\rm{V}}}}\text{O}$_2max_, *BLa* Blood lactate.

^a^Only 11 participants were analyzed due to technical difficulties.

*p < 0.05, **p < 0.01, ***p < 0.001: Indicates significant difference between endurance-trained and sprint-trained athletes at T_lim_v${{\dot{\rm{V}}}}\text{O}$_2max_ and T_lim_Vsub%95.

All athletes attained ≥  95%${{\dot{\rm{V}}}}\text{O}$_2max_ to calculate TA${{\dot{\rm{V}}}}\text{O}$_2max_ at T_lim_v${{\dot{\rm{V}}}}\text{O}$_2max_ and T_lim_Vsub%95 (Table 5). v${{\dot{\rm{V}}}}\text{O}$_2max_ and Vsub%95 were significantly higher among endurance-trained athletes (*p* ≤ 0.001). However, sprint-trained athletes ran at these speeds for longer duration and hence T_lim_ was significantly different compared to ET athletes (*p* = 0.030). No significant differences were determined between both cohorts for TA${{\dot{\rm{V}}}}\text{O}$_2max_ T_lim_${{\dot{\rm{V}}}}\text{O}$_2max_, and BLa at T_lim_v${{\dot{\rm{V}}}}\text{O}$_2max_ (*p* = 0.164) and T_lim_Vsub%95 (*p* = 0.264) (Table 5). Similar results were also calculated for T_lim_${{\dot{\rm{V}}}}\text{O}_{\text{2maxconverted}}$ (sprint-trained: 167.98 ± 52.28 s; endurance-trained: 125.75 ± 76.28 s, *p* = 0.171).

**Table 5:** Maximal speed of endurance-trained and sprint-trained athletes.

Variables	Endurance-trained	Sprint-trained
N	12	8
MS (km·h^−1^)	29.26 ± 1.33	35.21 ± 1.90***
Distance_MS_ (m)	36.29 ± 3.08	41.05 ± 3.14**
BLa_50 m_ (mmol·L^-1^)	4.16 ± 0.83	5.53 ± 1.17**
50 m (s)	7.38 ± 0.45	6.38 ± 0.43***

Values are in means ± SD.

*MS* Maximal speed, *Distance*_*MS*_ Distance at which MS determined, *BLa*_*50 m*_ Peak post-exercise blood lactate after 50 m sprint run test.

**p < 0.01, ***p < 0.001: Indicates significant difference between endurance-trained and sprint-trained athletes.

Mean CS (endurance-trained: 14.95 ± 1.40 km·h^−1^; sprint-trained: 11.52 ± 0.80 km·h^−1^, *p* < 0.001) was significantly higher while ADC (endurance-trained: 221.60 ± 57.74 m; sprint-trained: 313.43 ± 139.74 m,* p* < 0.05) was significantly lower in endurance-trained athletes compared to strength-trained athletes. MAS range was between 15.37 ± 1.57 km·h^−1^ (~ υΔ50) and 16.25 ± 1.64 km·h^−1^ (~ υΔ50 + 5%v${{\dot{\rm{V}}}}\text{O}$_2max_) among endurance-trained athletes and between 12.42 ± 0.81 km·h^−1^ (~ υΔ50) and 13.12 ± 0.85 km·h^−1^ (~ υΔ50 + 5%v${{\dot{\rm{V}}}}\text{O}$_2max_) among sprint-trained athletes.

Furthermore, endurance-trained athletes achieved significantly higher MAS (endurance-trained: 16.07 ± 1.58 km·h^−1^; sprint-trained: 12.77 ± 0.81 km·h^−1^, *p* ≤ 0.001; 95% CI [2.091, 4.515]) and E_MAS_ (endurance-trained: 52.87 ± 5.35 ml·kg^−1^·min^−1^; sprint-trained: 46.42 ± 3.38 ml·kg^−1^·min^−1^, *p* = 0.005; 95% CI [2.182, 10.716]) at significantly shorter MAS_dur_ (endurance-trained: 678.59 ± 165.44 s; sprint-trained: 840.28 ± 164.97 s, *p* = 0.039; 95% CI [− 314.190, − 9.177]) compared to sprint-trained athletes.

MAS was also significantly correlated to ${{\dot{\rm{V}}}}\text{O}$_2max_ (r = 0.78, *p* < 0.001), v${{\dot{\rm{V}}}}\text{O}$_2max_ (r = 0.98, *p* < 0.001). In addition, MAS had comparatively higher correlations with vLT (MAS: r = 0.97, *p* ≤ 0.001; v${{\dot{\rm{V}}}}\text{O}$_2max_: r = 0.91, *p* < 0.01), CS (MAS: r = 0.99; v${{\dot{\rm{V}}}}\text{O}$_2max_: r = 0.93), 5000 m (MAS: r = − 0.95, *p* < 0.001; v${{\dot{\rm{V}}}}\text{O}$_2max_: r = − 0.92), T_lim_υΔ50 + 5%v${{\dot{\rm{V}}}}\text{O}$_2max_ (MAS: *r* = − 0.71, *p* < 0.05; v${{\dot{\rm{V}}}}\text{O}$_2max_: r = − 0.62) and Vsub%95 (MAS: *r* = 0.997, *p* < 0.001; v${{\dot{\rm{V}}}}\text{O}$_2max_: r = 0.98, *p* < 0.01) compared to v${{\dot{\rm{V}}}}\text{O}$_2max_. MAS predicted the 5000 m speed and vLT with high accuracy (5000 m speed: *R*^2^ = 0.90; vLT: *R*^2^ = 0.96, *p* < 0.001).

Sprint-trained athletes had significantly higher Maximal Speed (MS) (*p* < 0.001) and achieved this speed at a significantly longer distance (*p* = 0.003). Significant differences were also observed in E_MAnS_, 50 m sprint performance (*p* < 0.001), and peak post-exercise BLa (*p* = 0.005) in the 50 m sprint run test (Table 5).

## Limitations

In general, the present study had no gold standard technique to validate anaerobic techniques, which may be presented as one of the limitations. Although there are other anaerobic techniques, such as, cycling or jumping, these norms are activity specific and may not accurately predict the anaerobic energy of runners or athletes involved in running. The present investigation’s results could only be compared to a similar technique, Bundle’s et al.^21^ anaerobic speed reserve (AnSR). The comparison in results indicated a high correlation between both methods, which indicated that MAS may also predict accurate all-out run performances. However, the accuracy of MAS to categorize middle distance athletes was not reported. Also, the techniques used for MAS in this study was different from Bundle’s use of MAS and utilizing MAS in the RERI^18^ had a lower error for prediction. The backward validation with lower error in prediction values was the only way to validate MAS. In future, MAS could be used to validate other similar anaerobic techniques.

In addition, the effect of training on MAS was not determined. Perhaps for future studies, the effect of different types of training, such as sprint or endurance or a combination of both, on MAS can be studied. Therefore, extending the accuracy of MAS in significantly differentiating middle distance athletes may increase the sensitivity of the model to detect even small changes in energy.

## Discussion

The results from this study confirmed the hypothesis that MAS is more accurate to be measured at %v${{\dot{\rm{V}}}}\text{O}$_2max_ than at v${{\dot{\rm{V}}}}\text{O}$_2max_. The determination of MAS required a subtraction of T_lim_${{\dot{\rm{V}}}}\text{O}$_2max__converted_ at v${{\dot{\rm{V}}}}\text{O}$_2max_ from T_lim_Vsub%95. This equation eliminated the anaerobic energy contribution. The concept of this study is therefore unique as the MAS determination has very little anaerobic contribution and has revealed low errors in predicting performance timings^18^.

### Validation of MAS

MAS was obtained at 92.45 ± 1.47%v${{\dot{\rm{V}}}}\text{O}$_2max_ and 89.27 ± 3.56%v${{\dot{\rm{V}}}}\text{O}$_2max_ for endurance-trained and sprint-trained athletes respectively, confirming the hypothesis that MAS should be obtained at a percentage of v${{\dot{\rm{V}}}}\text{O}$_2max_ rather than at v${{\dot{\rm{V}}}}\text{O}$_2max_. Studies have determined higher anaerobic energy at T_lim_v${{\dot{\rm{V}}}}\text{O}$_2max_ that was verified by a non-significant difference between BLa at v${{\dot{\rm{V}}}}\text{O}$_2max_ (${\text{BLa}}_{{{\text{v} \dot{\rm{V}}}}\text{O}_{\text{2max}}}$) and BLa at ${{\dot{\rm{V}}}}\text{O}$_2max_ (${\text{BLa}}_{{{\dot{\rm{V}}}}\text{O}_{\text{2max}}}$)^1,2,21,26^. Similarly, no significant difference was found in anaerobic energy contribution between T_lim_100%v${{\dot{\rm{V}}}}\text{O}$_2max_ (15.1 mmol·L ^−1^), T_lim_120 (15.7 mmol·L^−1^) and T_lim_140 (15.1 mmol·L^−1^)^16^. T_lim_v${{\dot{\rm{V}}}}\text{O}$_2max_ (269 ± 77 s) was also significantly correlated (*r* = − 0.52, *p* < 0.05) to T_lim_120v${{\dot{\rm{V}}}}\text{O}$_2max_ (86 ± 25 s) and to the blood pH after T_lim_120%v${{\dot{\rm{V}}}}\text{O}$_2max_ (*r* = − 0.68, *p* < 0.05).

On the contrary, E_MAS_ in this study was obtained at MAS. ${{\dot{\rm{V}}}}\text{O}$_2_ at MAS (50.69 ± 4.69 ml·kg^−1^·min^−1^) was found to be at 96.08 ± 2.51%${{\dot{\rm{V}}}}\text{O}$_2max_, which was not significantly different from 95%${{\dot{\rm{V}}}}\text{O}$_2max_ (50.18 ± 5.19 ml·kg^−1^·min^−1^). As most athletes did not reach E_MAS_ at speeds of 14.10 km·h^−1^ which was just below MAS (14.64 km·h^−1^), MAS seems to be the minimal intensity of the slow component of ${{\dot{\rm{V}}}}\text{O}$_2_. Additionally, MAS in this study was obtained at 91.08 ± 2.97%v${{\dot{\rm{V}}}}\text{O}$_2max_ for total cohort, which was similar to other studies where most athletes achieved ${{\dot{\rm{V}}}}\text{O}$_2max_ at 91%v${{\dot{\rm{V}}}}\text{O}$_2max_^30^. It was found that endurance-trained athletes in their study (${{\dot{\rm{V}}}}\text{O}$_2max_ = 60.7 ml·kg^−1^·min^−1^, v${{\dot{\rm{V}}}}\text{O}$_2max_ = 20 km·h^−1^) achieved approximately 99%${{\dot{\rm{V}}}}\text{O}$_2max_ at 90%v${{\dot{\rm{V}}}}\text{O}$_2max_ (18.3 km·h^−1^)^31^. This is close to 92.45%v${{\dot{\rm{V}}}}\text{O}$_2max_ at MAS among endurance-trained athletes in the present investigation. These studies suggest that submaximal speed is sufficient for achieving an increase in ${{\dot{\rm{V}}}}\text{O}$_2max_ and should be used for training^32^. These findings support the validity of MAS, which is the minimal speed at which E_MAS_ is determined.

In addition, BLa_MAS_ in this study was significantly lesser than ${\text{BLa}}_{{{{\rm v}\dot{{\rm V}}{\rm O}}}2\max}$and ${\text{BLa}}_{{{\dot{{\rm V}}{\rm O}}}2\max}$. This could be due to the slow component of ${{\dot{\rm{V}}}}\text{O}$_2_ at a slower speed, which is directly related to the recruitment of less efficient fast twitch fibers^30^, anaerobic energy utilization, and to the intensity of exercise^33–35^. The decrement of anaerobic energy with increasing duration at T_lim_MAS compared to T_lim_v${{\dot{\rm{V}}}}\text{O}$_2max_ could lead to lower BLa_MAS_ compared to ${\text{BLa}}_{{{\rm v}\dot{{\rm V}}{\rm O}}}$_2max_. It was also determined that there was significant correlation between the slow component of ${{\dot{\rm{V}}}}\text{O}$_2_ with indices of anaerobic performance (WAnT’s peak power; r = 0.77, *p* < 0.01)^36^. Since there is an inverse relationship between TA${{\dot{\rm{V}}}}\text{O}$_2max_ and exercise intensity^37^, TA${{\dot{\rm{V}}}}\text{O}$_2max_ would have been higher at T_lim_MAS as compared to T_lim_v${{\dot{\rm{V}}}}\text{O}$_2max_. E_MAS_ would have been attained in the later part of the run, which may minimize anaerobic energy contribution. This was shown in the present study and confirmed that MAS calculated was accurate.

### MAS between endurance-trained and sprint-trained athletes

This study also found that sprint-trained athletes had significantly lower MAS compared to endurance-trained athletes. This was evident in their vLT, ${{\dot{\rm{V}}}}\text{O}$_2max_, and v${{\dot{\rm{V}}}}\text{O}$_2max_ variables. Endurance training increases ${{\dot{\rm{V}}}}\text{O}$_2max_ by increasing cardiac stroke volume, blood volume, capillary density, and mitochondrial density in trained muscles^35^, allowing endurance-trained athletes to have higher ${{\dot{\rm{V}}}}\text{O}$_2max_, vLT, and v${{\dot{\rm{V}}}}\text{O}$_2max_ compared to sprint-trained athletes.

Additionally, MAS had comparatively higher significant correlations with CS, vLT, 5000 m, T_lim_υΔ50 + 5%v${{\dot{\rm{V}}}}\text{O}$_2max_, and Vsub%95 compared to v${{\dot{\rm{V}}}}\text{O}$_2max_. Furthermore, MAS was a stronger predictor of 5000 m and vLT. This was similar to a study conducted by Blondel, Berthoinm Billat & Lensel (2001), who also found significant correlations between 90%v${{\dot{\rm{V}}}}\text{O}$_2max_ and CS (r = 0.69,* p* < 0.05)^16^. Additional analysis found that there was a significant negative correlation with maximal speed reserve (MSR; difference between MS and CS; r = 0.79, *p* ≤ 0.001). This relationship is consistent with previous studies who found that endurance-trained athletes with lower MSR achieved vLT and CS at higher speeds and had lower MAnS compared to sprinters^16,38^. These findings support the utility of MAS in predicting performances in most running events and may suggest more accurate performance prediction at MAS rather than at v${{\dot{\rm{V}}}}\text{O}$_2max_.

## Conclusion

In conclusion, this study aimed to determine the intensity at which aerobic energy contribution is at maximal. MAS in this study was found to be at 92.45 ± 1.47%v${{\dot{\rm{V}}}}\text{O}$_2max_ for endurance trained athletes, 89.27 ± 3.56%v${{\dot{\rm{V}}}}\text{O}$_2max_ for sprint trained athletes, and 91.08 ± 2.97%v${{\dot{\rm{V}}}}\text{O}$_2max_ among the total cohort. This accurately represented E_MAS_ with minimal contribution from anaerobic energy sources, thus confirming the hypothesis that MAS is more accurate at %v${{\dot{\rm{V}}}}\text{O}$_2max_ rather than at v${{\dot{\rm{V}}}}\text{O}$_2max_. MAS for endurance-trained athletes were also significantly higher compared to sprint-trained athletes, indicating that MAS can differentiate between the types of athletes. Furthermore, MAS was found to significantly correlate with aerobic performance variables, and this suggest that submaximal speed is sufficient for training athletes. Regardless the profile of the individual, recreational athletes, collegiate athletes, elite athletes, coaches, and sports practitioners may utilize this MAS calculation to accurately derive the athlete’s individual main energy contribution source (anaerobic or aerobic energy source). Coaches may use their athletes’ MAS to prescribe training workouts that are specifically catered to them, which will predict an accurate sporting performance. Therefore, this new MAS framework demonstrates that the accurate calculation of MAS can accurately predict run performances at lower errors.^18^
